# Efficacy and Safety of Intravenous Thrombolysis Beyond 4.5 Hours in Ischemic Stroke: A Systematic Review and Meta-Analysis

**DOI:** 10.3390/diagnostics15141812

**Published:** 2025-07-18

**Authors:** Muhammad Ahmad, Chavin Akalanka Ranasinghe, Mais Omar Abu-Sa’da, Durga Prasad Bhimineni, Muhammed Ameen Noushad, Talal Warsi, Ahmad Mesmar, Munikaverappa Anjanappa Mukesh, Sagar K. Patel, Gabriel Imbianozor, Ali Mustansir Bhatty, Ahmad Alareed, Quratul Ain, Eeshal Zulfiqar, Mushood Ahmed, Raheel Ahmed

**Affiliations:** 1 Junior Clinical Fellow Geriatrics/Acute Medicine, Aneurin Bevan University Healthboard, Caerleon NP18 3XQ, UK; muhammad.ahmad5@wales.nhs.uk; 2 Department of Cardiology, Queen Elizabeth Hospital Birmingham, Birmingham B15 2GW, UK; akalanka1824@gmail.com; 3 RAKCOMs, RAK Medical Health Sciences University, Ras Al Khaimah 11172, United Arab Emirates; mais.20901036@rakmhsu.ac.ae; 4 Cardiology, West Suffolk NHS Foundation Trust, Bury St Edmunds, Suffolk IP33 2QZ, UK; durgaprasad.bheemineni@gmail.com; 5 Neurology, University Hospitals Plymouth NHS Trust, Plymouth PL6 8DH, UK; muhammedameen.noushad@nhs.net; 6 Cardiology, University Hospitals Plymouth NHS Trust, Plymouth PL6 8DH, UK; talal.warsi@nhs.net; 7 Cardiology Department, Sheikh Shakhbout Medical City, Abu Dhabi P.O. Box 11001, United Arab Emirates; a7mad-missmar@hotmail.com; 8 Internal Medicine, South Tyneside and Sunderland NHS Foundation Trust, Sunderland R4 7TP, UK; mukesh.anjanappa@nhs.net; 9 Gujarat Adani Institute of Medical Sciences, Bhuj 370001, Gujarat, India; patelsagar9799@gmail.com; 10 Royal Wolverhampton NHS Trust, Wolverhampton WV10 0QP, UK; imbianozorgabriel@gmail.com; 11 Hull University Teaching Hospital NHS Trust, Hull HU1 3SA, UK; ali.bhatty@nhs.net; 12 University Hospital Southampton NHS Foundation Trust, Southampton SO16 6YD, UK; ahmad.alareed@nhs.net; 13 Department of Medicine, Rotherham General Hospital, Rotherham S60 2UD, UK; quratul.ain3@nhs.net; 14 Department of Medicine, Dow University of Health Sciences, Karachi 75280, Pakistan; eeshalzulfiqar12@gmail.com; 15 Department of Medicine, Rawalpindi Medical University, Rawalpindi 46000, Pakistan; mushood07@gmail.com; 16 National Heart & Lung Institute, Imperial College London, London W12 0NN, UK; 17 Department of Cardiology, Royal Brompton Hospital, London SW3 6NP, UK

**Keywords:** ischemic stroke, thrombolysis, tenecteplase, alteplase, meta-analysis

## Abstract

**Background**: Intravenous thrombolysis (IVT) is the standard treatment for ischemic stroke within 4.5 h of symptom onset. However, a significant proportion of patients present beyond this window. This study aims to evaluate the efficacy and safety of IVT beyond the 4.5 h window in selected patients. **Methods**: A systematic literature search was conducted across PubMed, Cochrane Library, and Google Scholar from inception to April 2025. Odds ratios (ORs) with 95% confidence intervals (CIs) were pooled using a random-effects model. **Results**: A total of 12 RCTs were included, with 3236 patients. Compared to controls, IVT significantly improved excellent functional outcomes [OR: 1.40; 95% CI: 1.21–1.62] and good functional outcomes [OR: 1.26; 95% CI: 1.06–1.50] at 90 days. IVT also improved recanalization [OR: 2.47; 95% CI: 1.96–3.12], reperfusion [OR: 2.20; 95% CI: 1.26–3.84], and early neurological improvement [OR: 1.91; 95% CI: 1.12–3.26]. However, it was associated with a significantly higher risk of symptomatic intracranial hemorrhage (sICH) [OR: 2.17; 95% CI: 1.25–3.79], any ICH [OR: 1.49; 95% CI: 1.09–2.04], and type-II parenchymal hemorrhage (PH) [OR: 2.14; 95% CI: 1.19–3.83]. No significant difference was observed in systemic hemorrhage, 90-day all-cause mortality, 7-day mortality, or 90-day intervention-related mortality (*p* > 0.05). **Conclusions**: IVT beyond 4.5 h improves neurological outcomes in patients with ischemic stroke without increasing overall mortality or systemic bleeding, though it raises the risk of sICH, any ICH, and type-II PH. Further large RCTs are needed to confirm these findings and guide clinical practice.

## 1. Introduction

Ischemic stroke, which accounts for nearly 87% of all stroke cases, remains a leading cause of disability and mortality worldwide, responsible for approximately 3.29 million deaths annually [1,2]. In recent years, mechanical thrombectomy (MT) has significantly advanced the treatment of acute ischemic stroke and is now supported by a level 1a recommendation in clinical guidelines [3]. MT has shown substantial benefits even in extended time windows of up to 24 h or in cases with unknown onset, provided patients are carefully selected [4,5]. Despite this progress, only a small proportion of stroke patients receive thrombectomy due to the absence of large vessel occlusion or limited access to timely intervention. Access challenges persist even in high-income countries and are more pronounced in low- and middle-income settings [6]. Consequently, intravenous thrombolysis (IVT) continues to be the most widely implemented reperfusion therapy for acute ischemic stroke worldwide.

Current guidelines recommend IVT treatment within 4.5 h of stroke onset [7]. However, a significant proportion of patients present beyond this window, limiting their eligibility for IVT. Globally, fewer than 5% of patients with acute ischemic stroke receive IVT within the recommended time window, highlighting a significant treatment gap [8]. Limited data exist on outcomes beyond the conventional window; therefore, several recent trials have explored whether IVT may still be beneficial in selected patients beyond 4.5 h, yielding conflicting results. The Echoplanar Imaging Thrombolytic Evaluation Trial (EPITHET), one of the earliest randomized, double-blind, placebo-controlled trials, evaluated intravenous alteplase administered between 3 and 6 h after symptom onset [9]. They demonstrated that alteplase significantly improved reperfusion and was associated with better neurological and functional outcomes compared to placebo. Similarly, the most recent Extending the Time Window for Thrombolysis in Posterior Circulation Stroke without Early CT Signs (EXPECTS) trial, found that in patients experiencing mostly mild posterior circulation ischemic stroke who were ineligible for thrombectomy, treatment with alteplase administered 4.5 to 24 h after stroke onset produced a higher rate of functional independence compared to standard care, without an increased risk of symptomatic intracranial hemorrhage (sICH) [10]. Similar trials have been conducted with tenecteplase. The Tenecteplase Reperfusion Therapy in Acute Ischemic Cerebrovascular Events–III (TRACE III) trial suggested that tenecteplase may achieve comparable or improved outcomes, though with a higher incidence of sICH [11].

Günkan et al. conducted a meta-analysis of eight trials, showing that IVT beyond the 4.5 h window improved functional outcomes but was associated with an increased risk of sICH [12]. Since then, several new trials have been published, offering further insights into the efficacy and safety of thrombolysis in extended time windows. Therefore, we performed this meta-analysis to systematically assess the efficacy and safety of IVT beyond 4.5 h in ischemic stroke patients, incorporating direct comparisons between alteplase and tenecteplase and including the most recent evidence available.

## 2. Methods

This systematic review and meta-analysis followed the guidelines established by Preferred Reporting Items for Systematic Review and Meta-Analysis (PRISMA) [13]. The study protocol was registered with PROSPERO (registration number: CRD420251058378). No ethical approval was required since the included studies were publicly available and all patient information was de-identified.

### 2.1. Data Sources and Search Strategy

A comprehensive search was conducted by two independent researchers (EZ and MA) across databases including PubMed, Google Scholar, and the Cochrane Library, from their inception until April 2025. The detailed search strategies are reported in Table S1. Additionally, we manually screened the reference lists of selected articles, previous reviews, and meta-analyses to ensure no relevant studies were missed in the initial search.

### 2.2. Study Selection and the Eligibility Criteria

All articles retrieved from the electronic search were imported into the Rayyan software, https://www.rayyan.ai (accessed on 13 April 2025), where duplicates were sought and removed. The remaining studies were independently reviewed by two investigators (TW and AR) by titles and abstracts, followed by a review of the full texts of the articles. Any disagreements were resolved by a third author (EZ).

Studies were included in our meta-analysis if they met the following criteria: (1) they were randomized controlled trials (RCTs); (2) enrolled patients had ischemic stroke and received IVT beyond 4.5 h of symptom onset; (3) they reported at least one of the following outcomes at 90 days: excellent functional outcome, defined as a modified Rankin Scale (mRS) score 0–1, good functional outcome (mRS score 0–2), all-cause mortality, symptomatic intracranial hemorrhage (sICH), and systemic hemorrhage.

The exclusion criteria were as follows: (1) retrospective studies, reviews, editorials, or meta-analyses; (2) enrolled patients who received IVT within the 4.5 h window; and (3) published in a language other than English.

### 2.3. Data Extraction, Outcomes, and Quality Assessment

Two investigators (MA and MO) autonomously extracted data from the selected studies. The extracted information included the following: trial name, year of publication, type of intervention, sample size, patient age, sex, baseline mRS score, National Institutes of Health Stroke Scale (NIHSS) score, time window to treatment, site of vascular occlusion, whether any patients underwent endovascular thrombectomy (EVT), along with the number of such cases when applicable, and outcome measures.

The primary efficacy outcome was an excellent functional outcome, defined as an mRS score 0–1 at 90 days. Secondary efficacy outcomes included the following: a good functional outcome (mRS score 0–2 at 90 days), recanalization, reperfusion, and early neurological improvement. The primary safety outcome was symptomatic intracranial hemorrhage (sICH). Secondary safety outcomes included the following: 90-day all-cause mortality, any intracranial hemorrhage (ICH), type-II parenchymal hemorrhage (PH), systemic hemorrhage, 7-day all-cause mortality, and 90-day intervention-related mortality. The definitions used for these outcomes across the included studies are detailed in Table S2.

To assess the quality of the included RCTs, the Cochrane Risk of Bias tool (RoB 2.0) was used [14]. This tool assesses bias across five domains: randomization, deviation from the intended intervention, missing outcome data, measurement of the outcome, and selection of the reported result. Studies were rated as having low risk, some concerns, or high risk of bias in each domain. Traffic-light and summary plots were generated using the Robvis tool [15].

### 2.4. Statistical Analysis

Statistical analysis was performed using RevMan, Version 5.4 (Nordic Cochrane Center, Copenhagen, Denmark). Odds ratios (ORs) were pooled along with their corresponding 95% CIs for each clinical outcome. The DerSimonian–Laird random effects model was used to account for inter-study heterogeneity [16]. Forest plots were created to visualize the results. Heterogeneity across studies was evaluated using Higgins I^2^, and a value of I^2^ = 25–50% was considered mild, 50–75% as moderate, and I^2^ > 75% as severe [17]. For the primary outcomes, we conducted subgroup analysis based on the thrombolytic agent used, whether patients received EVT, and by the imaging technique used. Publication bias was assessed using funnel plot analysis and Egger’s regression test [18]. A *p*-value of <0.05 was considered statistically significant in all cases.

### 2.5. Certainty of Evidence Assessment

We assessed the certainty of evidence for each outcome using the GRADE framework, which rates the quality of evidence as high, moderate, low, or very low [19,20]. Ratings were based on factors such as risk of bias, inconsistency, imprecision, indirectness, and publication bias (Table S3).

## 3. Results

### 3.1. Study Selection

**Figure 1:**
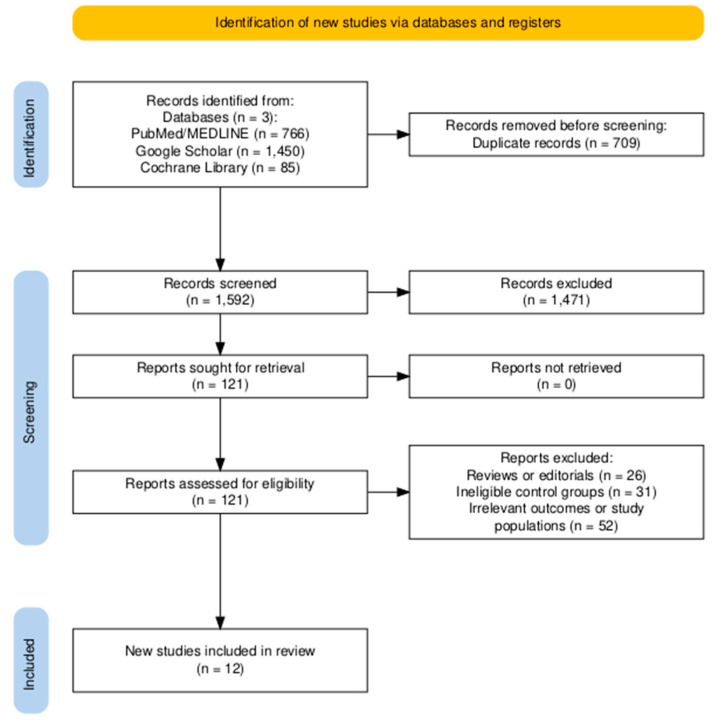
PRISMA Flow Diagram.

A total of 2301 records were retrieved from various databases. Following the removal of 709 duplicates, 1592 unique records remained for title and abstract screening. Of these, 1471 were excluded, and the full texts of 121 articles were retrieved for screening. After full-text review, 12 studies met the inclusion criteria [10,11,21,22,23,24,25,26,27,28,29,30]. The PRISMA flowchart (Figure 1) summarizes the screening and study selection process.

### 3.2. Study and Patient Characteristics

All included studies were RCTs published between 2014 and 2025. Alteplase was used as the thrombolytic agent in six trials, while tenecteplase was employed in the remaining six. Among the 12 included RCTs, three enrolled patients received EVT. The pooled analysis included 3236 patients, with 1634 in the intervention group and 1602 in the control group. Of these, 1965 (60.7%) were males and 1271 (39.3%) were females, with a mean age of 68.9 (SD ± 12.1). The baseline mRS score of 0–1 was reported in 2403 patients (1219 in the intervention group and 1184 in the control group). Detailed baseline characteristics of the included studies and patients are provided in Table 1.

### 3.3. Results of Quality Assessment

Risk of bias assessment using the RoB 2.0 tool indicated a low risk in seven RCTs, while five trials raised some concerns. The details of bias assessment are provided in Figures S1 and S2.

### 3.4. Efficacy Outcomes

Excellent functional outcome (mRS score 0–1)

**Figure 2:**
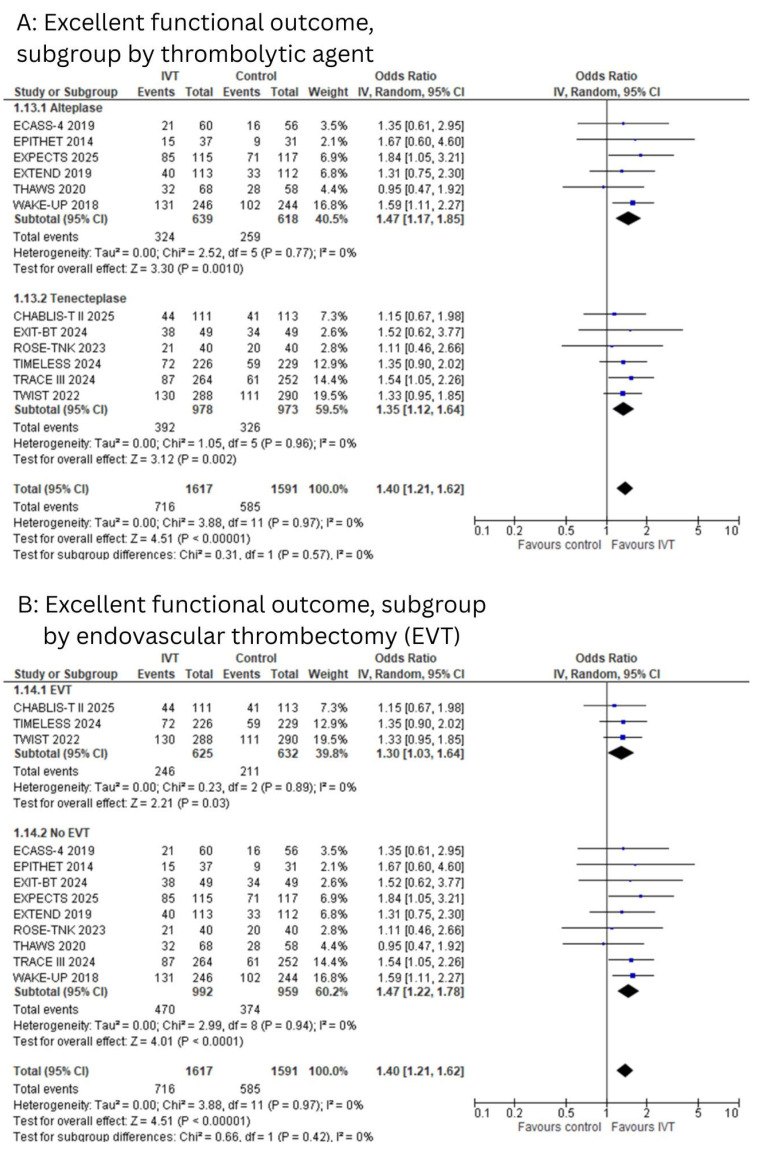
(**A**): Forest plot for excellent functional outcomes, subgroups grouped by type of thrombolytic agent. (**B**): Forest plot for excellent functional outcomes, subgroups grouped by EVT status.

All studies reported excellent functional outcomes, defined as an mRS score of 0–1 at 90 days. The pooled analysis demonstrated a statistically significant improvement in excellent functional outcomes among patients who received IVT compared to controls (OR: 1.40; 95% CI: 1.21 to 1.62; I^2^ = 0%; *p* < 0.00001, Figure 2A,B). Egger’s test showed no significant risk of publication bias (*p* = 0.50).

The subgroup analysis based on the type of thrombolytic agent revealed a significant benefit for both alteplase (OR: 1.47; 95% CI: 1.17 to 1.85; I^2^ = 0%; *p* = 0.001) and tenecteplase (OR: 1.35; 95% CI: 1.12 to 1.64; I^2^ = 0%; *p* = 0.002) compared to controls, with no significant subgroup difference between the two agents (*p* = 0.57, Figure 2A).

When stratified by EVT status, IVT was associated with a significantly higher rate of excellent outcomes in both the EVT (OR: 1.30; 95% CI: 1.03 to 1.64; I^2^ = 0%; *p* = 0.03) and non-EVT groups (OR: 1.47; 95% CI: 1.22 to 1.78; I^2^ = 0%; *p* < 0.0001), with no significant difference between subgroups (*p* = 0.42, Figure 2B).

In the subgroup analysis by imaging technique, IVT significantly improved excellent functional outcomes in patients selected using either DWI-FLAIR mismatch (OR: 1.39; 95% CI: 1.03 to 1.87; I^2^ = 0%; *p* = 0.03) or MRI/CT perfusion imaging (OR: 1.36; 95% CI: 1.10 to 1.70; I^2^ = 0%; *p* = 0.005), compared to controls. There was no statistically significant subgroup difference between the two imaging modalities (*p* = 0.94, Figure S3).

Good functional outcome (mRS score 0–2)

**Figure 3:**
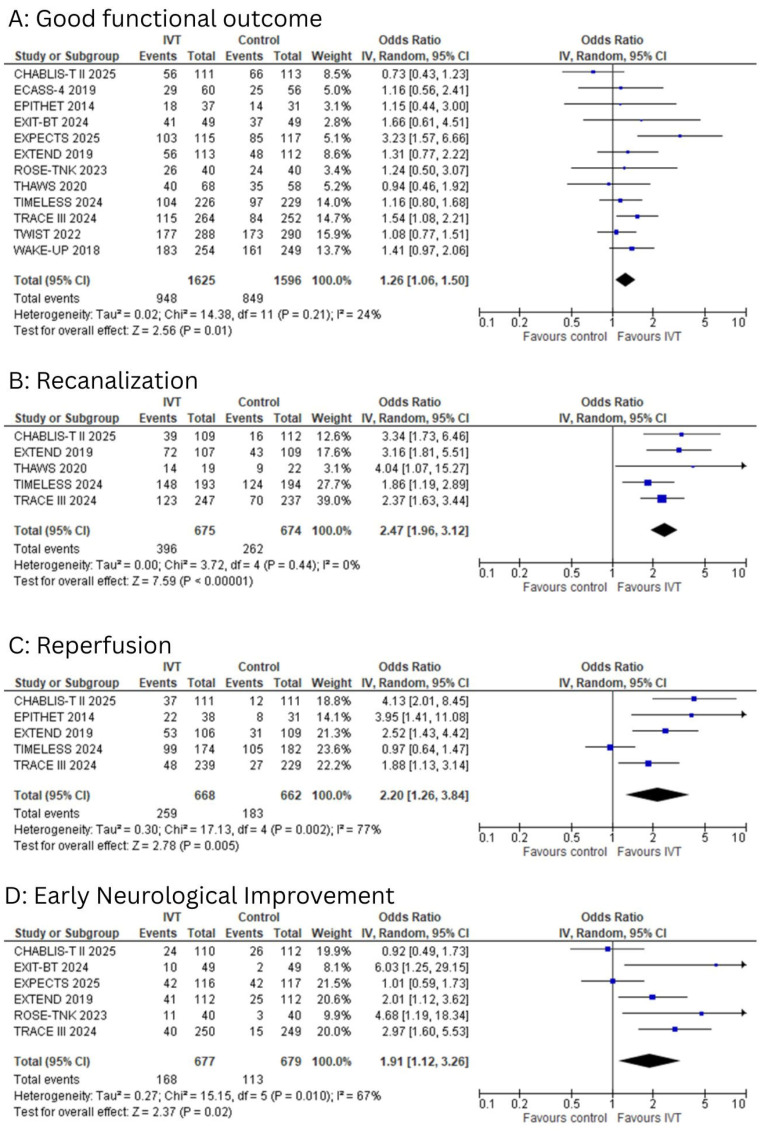
(**A**): Forest plot for good functional outcomes. (**B**): Forest plot for recanalization. (**C**): Forest plot for reperfusion. (**D**): Forest plot for early neurological improvement.

All studies reported good functional outcomes, defined as an mRS score of 0–2 at 90 days. The pooled analysis demonstrated a statistically significant improvement in good functional outcomes among patients who received IVT compared to controls (OR: 1.26; 95% CI: 1.06 to 1.50; I^2^ = 24%; *p* = 0.01, Figure 3A). Egger’s test showed no significant risk of publication bias (*p* = 0.74).

Recanalization

Five studies reported the outcome of recanalization. A significant association was observed between IVT and improved recanalization compared to controls (OR: 2.47, 95% CI: 1.96–3.12, I^2^ = 0%, *p* < 0.00001, Figure 3B).

Reperfusion

Five studies reported the outcome of reperfusion. A significant association was observed between IVT and improved reperfusion compared to controls (OR: 2.20, 95% CI: 1.26–3.84, I^2^ = 77%, *p* = 0.005, Figure 3C). Significant heterogeneity was detected among the included studies (I^2^ = 77%).

Early Neurological Improvement

Six studies reported the outcome of early neurological improvement. A significant association was observed between IVT and improved early neurological outcomes compared to controls (OR: 1.91, 95% CI: 1.12–3.26, I^2^ = 67%, *p* = 0.02, Figure 3D). Moderate heterogeneity was detected among the included studies (I^2^ = 67%).

### 3.5. Safety Outcomes

Symptomatic intracranial hemorrhage (sICH)

**Figure 4:**
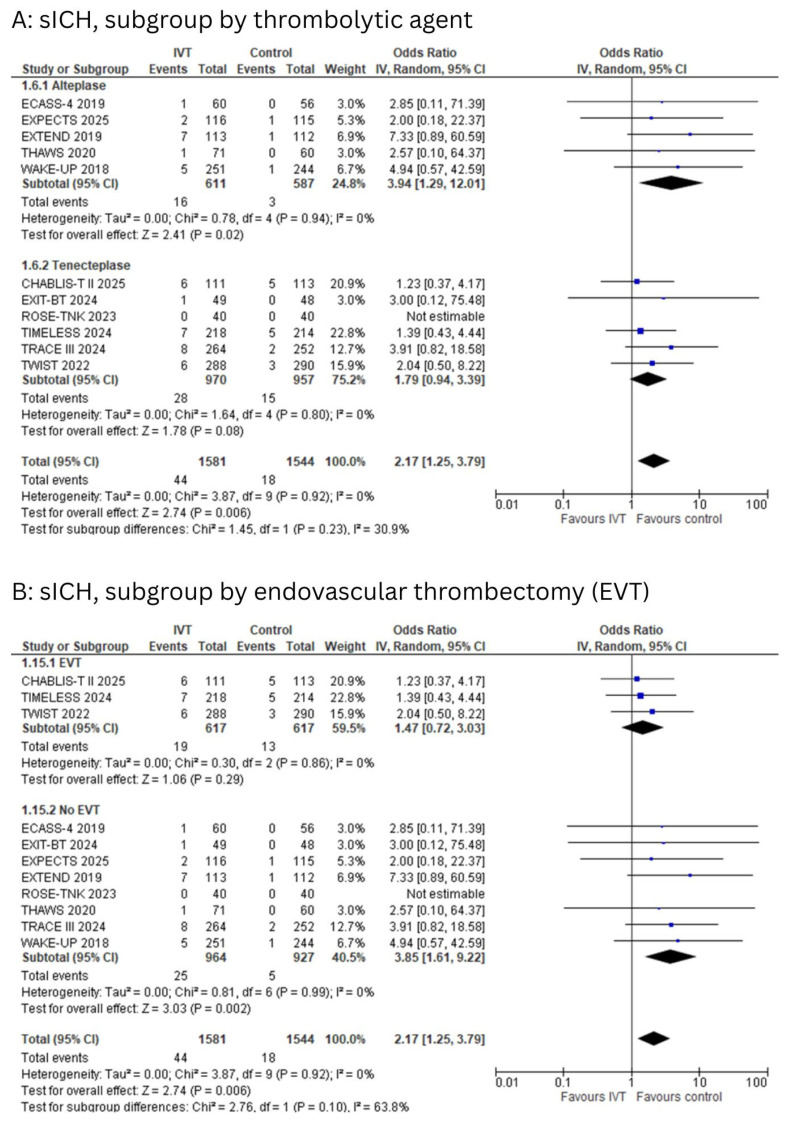
(**A**): Forest plot for symptomatic intracranial hemorrhage (sICH), subgroups grouped by type of thrombolytic agent. (**B**): Forest plot for symptomatic intracranial hemorrhage (sICH), subgroups grouped by EVT status.

Eleven studies reported symptomatic intracranial hemorrhage (sICH). The pooled analysis showed a statistically significant increase in sICH among patients who received IVT compared to controls (OR: 2.17; 95% CI: 1.25 to 3.79; I^2^ = 0%; *p* = 0.006, Figure 4A,B). Egger’s test showed no significant risk of publication bias (*p* = 0.09).

The subgroup analysis based on the type of thrombolytic agent revealed a significantly increased risk with alteplase (OR: 3.94; 95% CI: 1.29 to 12.01; I^2^ = 0%; *p* = 0.02), while the association was not statistically significant for tenecteplase (OR: 1.79; 95% CI: 0.94 to 3.39; I^2^ = 0%; *p* = 0.08), with no significant subgroup difference between the two agents (*p* = 0.23, Figure 4A).

When stratified by EVT status, IVT was associated with a significantly higher risk of sICH in the non-EVT group (OR: 3.85; 95% CI: 1.61 to 9.22; I^2^ = 0%; *p* = 0.002), whereas the association was not statistically significant in the EVT group (OR: 1.47; 95% CI: 0.72 to 3.03; I^2^ = 0%; *p* = 0.29), with no significant difference between subgroups (*p* = 0.10, Figure 4B).

In the subgroup analysis by imaging technique, IVT was associated with an increased, though not statistically significant, risk of sICH in both DWI-FLAIR mismatch (OR: 4.04; 95% CI: 0.67 to 24.19; I^2^ = 0%; *p* = 0.13) and MRI/CT perfusion-selected patients (OR: 2.00; 95% CI: 1.01 to 3.97; I^2^ = 0%; *p* = 0.05). The test for subgroup differences was not statistically significant (*p* = 0.47, Figure S4).

The 90-day all-cause mortality

**Figure 5:**
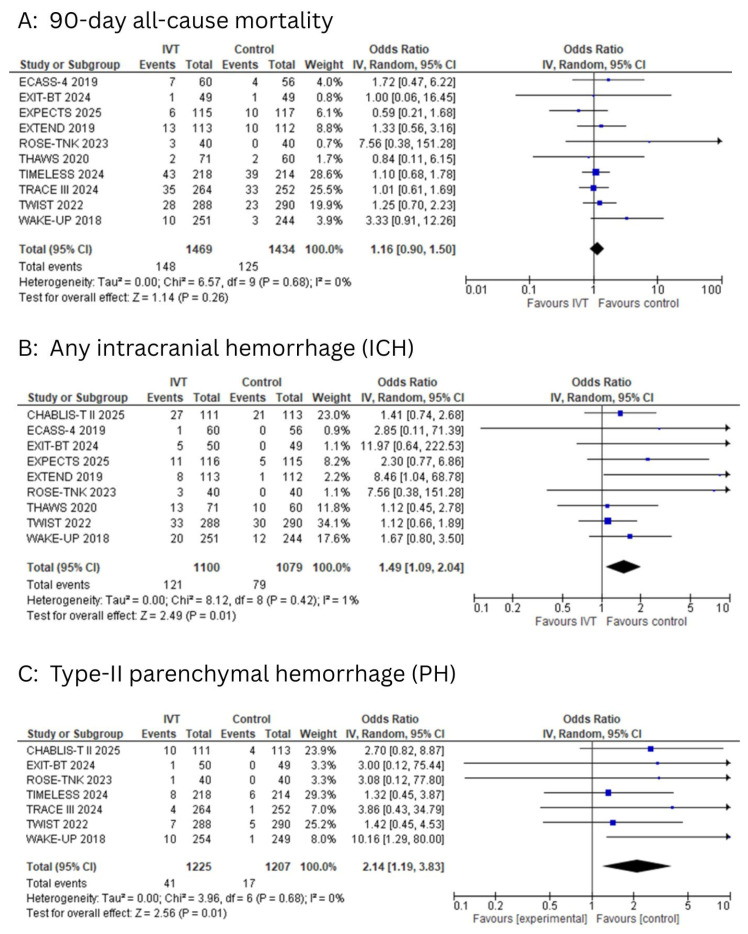
(**A**): Forest plot for 90-day all-cause mortality. (**B**): Forest plot for any intracranial hemorrhage (ICH). (**C**): Forest plot for type-II parenchymal hemorrhage (PH).

Ten studies reported the 90-day all-cause mortality. The pooled analysis demonstrated no statistically significant difference in mortality between patients who received IVT and those in the control group (OR: 1.16; 95% CI: 0.90 to 1.50; I^2^ = 0%; *p* = 0.26, Figure 5A). Egger’s test showed no significant risk of publication bias (*p* = 0.28).

Any intracranial hemorrhage (ICH)

Nine studies reported the outcome of ICH. The pooled analysis demonstrated a significantly higher risk of ICH in the IVT group compared to the control group (OR: 1.49; 95% CI: 1.09 to 2.04; I^2^ = 1%; *p* = 0.01; Figure 5B).

Type-II parenchymal hemorrhage (PH)

Seven studies reported the outcome of type-II PH. IVT was associated with a significantly higher risk of type-II PH compared to the control group (OR: 2.14; 95% CI: 1.19 to 3.83; I^2^ = 0%; *p* = 0.01; Figure 5C).

Systemic hemorrhage

**Figure 6:**
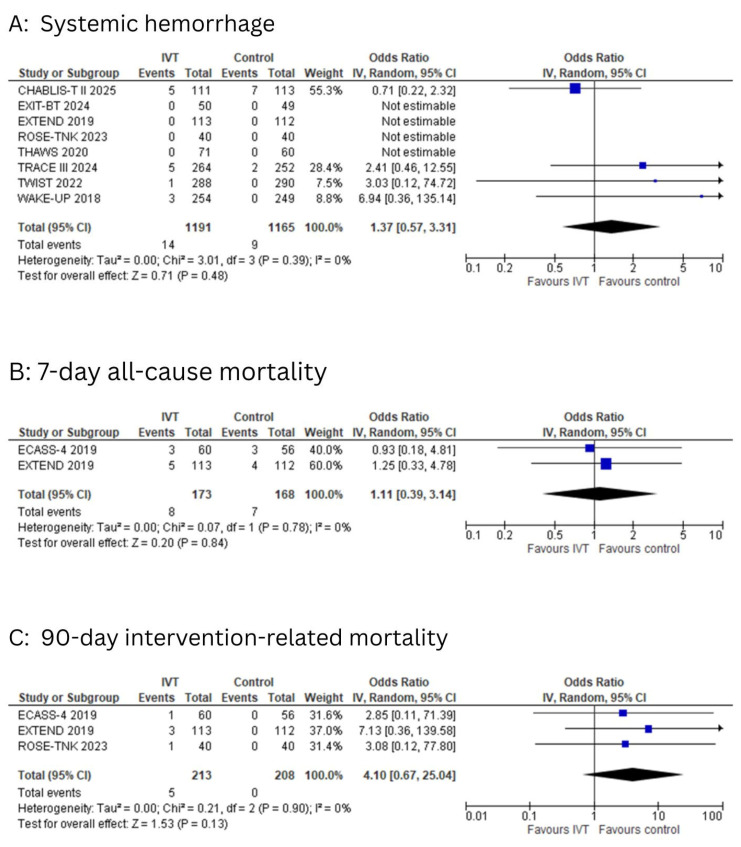
(**A**): Forest plot for systemic hemorrhage. (**B**): Forest plot for 7-day all-cause mortality. (**C**): Forest plot for 90-day intervention-related mortality.

Eight studies reported the outcome of systemic hemorrhage. The pooled analysis demonstrated no statistically significant difference in systemic hemorrhage between patients who received IVT and those in the control group (OR: 1.37; 95% CI: 0.57 to 3.31; I^2^ = 0%; *p* = 0.48, Figure 6A).

The 7-day all-cause mortality

Two studies reported the outcome of 7-day all-cause mortality. No significant association was observed between the two groups (OR: 1.11; 95% CI: 0.39 to 3.14; I^2^ = 0%; *p* = 0.84, Figure 6B).

The 90-day intervention-related mortality

Three studies reported the outcome of 90-day intervention-related mortality. No significant association was observed between the two groups (OR: 4.10; 95% CI: 0.67 to 25.04; I^2^ = 0%; *p* = 0.13, Figure 6C).

### 3.6. Leave-One-Out Sensitivity Analysis

A leave-one-out sensitivity analysis was performed for outcomes demonstrating at least moderate heterogeneity (I^2^ ≥ 50%). For the outcome of reperfusion, excluding TIMELESS [28] reduced the heterogeneity to 21%, and the pooled estimates showed significantly improved reperfusion in the IVT group compared to controls (OR: 2.65; 95% CI: 1.84 to 3.83; I^2^ = 21%; *p* < 0.00001, Figure S5).

For early neurological improvement, excluding either the EXPECTS [10] or TRACE III [11] trials individually reduced the heterogeneity to 63%. When EXPECTS was excluded, the pooled effect remained statistically significant (OR: 2.27; 95% CI: 1.24 to 4.14; I^2^ = 63%; *p* = 0.008, Figure S6). However, excluding TRACE III rendered the pooled effect non-significant (OR: 1.69; 95% CI: 0.95 to 3.04; I^2^ = 63%; *p* = 0.08, Figure S7).

Funnel plots for each pooled outcome with ≥10 studies are provided in Figures S8–S11.

## 4. Discussion

In this systematic review and meta-analysis of 12 RCTs comprising 3236 patients, we evaluated the efficacy and safety of IVT in patients with ischemic stroke. The pooled analysis revealed that IVT significantly improved both excellent (mRS 0–1) and good (mRS 0–2) functional outcomes at 90 days, with consistent benefits observed across subgroups defined by the thrombolytic agent (alteplase or tenecteplase) and EVT status. Additionally, IVT was associated with significantly higher odds of successful recanalization, reperfusion, and early neurological improvement compared to controls. However, IVT was also associated with an increased risk of sICH, especially among patients who did not undergo EVT and those treated with alteplase. IVT significantly increased the risk of any ICH and type-II PH but did not significantly impact the risk of systemic hemorrhage. Furthermore, no statistically significant differences were observed between the IVT and control groups in terms of 90-day all-cause mortality, 7-day mortality, or 90-day intervention-related mortality. 

Our pooled analysis demonstrated that IVT beyond 4.5 h significantly improved both excellent (mRS 0–1) and good (mRS 0–2) functional outcomes at 90 days. This finding aligns with results from key trials and meta-analyses conducted in the extended time window. An individual patient-level meta-analysis of the EXTEND and EPITHET trials showed that intravenous alteplase significantly improved functional outcomes across all late-window subgroups (4.5–6 h, 6–9 h, and wake-up strokes), although the benefit for excellent outcomes was not statistically significant in the 6–9 h range [31]. Similarly, another patient-level meta-analysis of three RCTs demonstrated an overall benefit of alteplase [32]. However, as nearly half the participants had wake-up strokes, the sample sizes for the other two time windows were comparatively limited. More recent trials have extended this evidence to tenecteplase. For example, the TRACE III trial demonstrated that tenecteplase resulted in a significantly higher rate of excellent functional outcomes (mRS 0–1) at 90 days (33.0%) compared to standard medical care (24.2%) [11]. Importantly, our subgroup analyses showed that benefits persisted across thrombolytic agents (both alteplase and tenecteplase) and irrespective of whether patients received EVT. These findings align with a recent meta-analysis of trials that excluded planned thrombectomy, which reported improved rates of excellent (mRS 0–1, 43.9%) and good (mRS 0–2, 36%) functional outcomes with extended-window IVT [12]. In their analysis, the direction of benefit was consistent across different imaging modalities and thrombolytic agents. Notably, trials that employed perfusion imaging or tested tenecteplase showed numerically greater odds of excellent functional outcomes compared to their respective counterparts. Nonetheless, important methodological differences across the included trials must be considered. Most alteplase trials limited treatment to within 9 h or selected patients using DWI-FLAIR mismatch, whereas several tenecteplase trials extended the time window up to 24 h and often included patients with anterior circulation strokes. Additionally, only certain tenecteplase trials allowed the use of EVT, while none of the alteplase trials did. This introduces potential confounding in the subgroup analysis by EVT status, as differences in thrombolytic agent use may have influenced the observed effects.

Recent studies have subsequently focused on comparing tenecteplase and alteplase for thrombolysis. Tenecteplase offers several pharmacological and practical advantages, including greater fibrin specificity, a longer half-life, and the convenience of single-bolus administration, which facilitates faster and simpler delivery and may improve door-to-needle times in clinical practice [33,34,35,36]. Multiple trials and meta-analyses have demonstrated that tenecteplase at the 0.25 mg/kg dose is non-inferior to alteplase in achieving good or excellent functional outcomes, with some studies suggesting a modest advantage [37,38,39,40]. It has been associated with higher rates of early neurological improvement and improved early reperfusion compared to alteplase [41,42]. Safety outcomes also favor tenecteplase, with large studies and analyses indicating similar or lower rates of sICH, and comparable or slightly reduced all-cause mortality at three months [37,43]. In trials enrolling patients within 4.5 h of symptom onset, tenecteplase at 0.25 mg/kg has consistently shown comparable efficacy to alteplase. The ATTEST-2 non-inferiority trial demonstrated equivalent 90-day mRS outcomes and similar rates of mortality and sICH in both groups, while the NOR-TEST trial, which primarily enrolled patients with mild strokes, found no significant difference in excellent outcomes [44,45]. A meta-analysis of 3707 patients confirmed no significant differences in excellent or good recovery [46]. Conversely, higher doses of tenecteplase (e.g., 0.4 mg/kg) have shown worse safety and efficacy, as reflected in the early termination of NOR-TEST 2A, reinforcing the preference for the 0.25 mg/kg dose [47]. In the extended time window, direct comparisons between tenecteplase and alteplase are lacking.

Consistent with improved clinical outcomes, IVT in our meta-analysis was associated with higher rates of vessel recanalization, reperfusion, and early neurological improvement. IVT remains an important component of acute ischemic stroke management, with the primary goal of restoring cerebral blood flow (reperfusion) by dissolving the occluding thrombus (recanalization). When administered prior to mechanical thrombectomy, IVT has been shown to increase rates of pre-interventional reperfusion by approximately 6–7% compared to thrombectomy alone, even in patients with large vessel occlusions and immediate access to endovascular therapy [48,49]. This early reperfusion may reduce the need for further endovascular intervention and support more rapid and effective recanalization. Pooled data from the EXTEND and EPITHET trials demonstrated that alteplase significantly increased major reperfusion rates compared to placebo (51% vs. 28%) in the 4.5–9 h and wake-up stroke windows, with similar benefits across different time strata [31]. Although data on tenecteplase in extended windows remain limited, emerging evidence is encouraging. In the TIMELESS trial, intravenous tenecteplase administered between 4.5 and 24 h in patients with large vessel occlusions resulted in higher rates of complete recanalization at 24 h compared to no thrombolysis [28]. Furthermore, the CHABLIS-T II trial demonstrated that, in Chinese patients with large or medium vessel occlusions, tenecteplase administered between 4.5 and 24 h after stroke onset significantly improved outcomes, with major reperfusion and recanalization rates markedly higher than those achieved with standard medical therapy alone (33.3% vs. 10.8% and 35.8% vs. 14.3%, respectively) [30]. This trial also reported comparable rates of early neurological improvement between the two groups. However, other studies have shown that patients treated with IVT beyond 4.5 h, particularly those selected using perfusion imaging, may experience increased rates of early neurological improvement [11,12].

We observed that IVT beyond 4.5 h was associated with a significantly increased risk of sICH. Subgroup analyses showed this risk was significant with alteplase but not with tenecteplase, and was greater in patients who did not receive EVT. As the time from stroke onset increases, irreversible brain injury worsens, compromising the blood–brain barrier and weakening cerebral vessels. The diminishing volume of salvageable tissue means thrombolysis in infarcted areas is more likely to cause hemorrhage than functional recovery. This risk is particularly pronounced in patients with extensive early ischemic changes or poor collateral circulation [50,51]. The prior literature on late-window IVT has consistently reported elevated sICH rates, especially in patients who did not undergo thrombectomy [12]. A meta-analysis by Al-Janabi et al. reported that sICH occurred in 2.9% of patients treated with IVT beyond the 4.5 h window, compared to 0.8% in controls [52]. Subgroup analysis revealed that the increased hemorrhagic risk was primarily confined to patients receiving IVT alone, with no significant rise in sICH among those who underwent bridging therapy with EVT, while the risk was significant in the IVT-only group. In addition to sICH, IVT beyond 4.5 h also increased the odds of any ICH and of type-II PH. This is expected given the above, and aligns with prior reports indicating that larger infarct volumes, particularly those that evolve gradually over several hours, are more susceptible to hemorrhagic transformation following thrombolysis. For example, both WAKE-UP and EXTEND noted higher total hemorrhage rates in the treatment arm, although it was often asymptomatic [22,24]. Similarly, prior meta-analyses of late IVT have shown elevated odds of total ICH and PH2 with thrombolysis [12]. Notably, these increases did not translate into higher death rates, suggesting that some hemorrhages were limited or clinically tolerated. We did not observe any increase in systemic bleeding outside the brain with IVT, in line with the established understanding that alteplase/tenecteplase have a much higher propensity for intracranial than extracranial hemorrhage when used in stroke [12]. Existing evidence indicates that, although IVT increases hemorrhage risk, it does not worsen survival. Consistent with the standard-window experience, the net effect on mortality in carefully selected patients appears neutral [12,32,52].

This is the largest and most comprehensive meta-analysis to date that directly evaluates the safety and efficacy of IVT in the extended time window for patients with acute ischemic stroke. Previous meta-analyses were limited by the smaller number of trials and the exclusion of patients undergoing EVT, despite EVT being a key component of modern stroke care. We performed a rigorous literature search and included the latest trials assessing both alteplase and tenecteplase beyond 4.5 h. Subgroup analyses were conducted to evaluate the influence of the thrombolytic agent and EVT status on primary outcomes. These findings have important clinical implications, supporting the role of extended-window IVT in appropriately selected patients based on advanced imaging criteria. Future trials should focus on refining patient selection criteria, evaluating the most effective dosing of thrombolytics, and directly comparing alteplase and tenecteplase in the late time window to further improve safety and efficacy.

### Limitations

Several limitations of our analysis should be acknowledged. First, this was a study-level rather than a patient-level meta-analysis, which restricts our ability to account for individual-level confounders and limits our capacity to assess the impact of missing data or the imputation strategies used in the original trials. Secondly, significant heterogeneity was observed in the outcomes of reperfusion and early neurological improvement, which may be attributed to variations in baseline patient characteristics, differences in inclusion criteria, thrombolytic agents used, time from last known well, and definitions of endpoints across the included studies. While we performed subgroup analyses by thrombolytic agent, EVT status, and imaging technique, additional subgroup analyses based on time intervals and stroke severity were not feasible due to inconsistent reporting of data. Although 12 RCTs were included, many were relatively small or terminated early, for instance, the WAKE-UP trial stopped at 63% of its intended sample size, which may limit the accuracy and generalizability of our findings. The analysis was restricted to 90-day outcomes, as longer-term data beyond three months were infrequently reported. Moreover, there was also variation in standard medical care across studies, with some trials administering only a placebo to control groups or not specifying whether standard care was provided (Table S4), which may have influenced outcomes. In addition, our analysis relied on ORs, which are widely used and align with prior meta-analyses. However, ORs may overestimate the magnitude of benefit when outcomes are common, and the lack of absolute risk estimates may limit clinical interpretability. Despite these constraints, the consistency of benefit across multiple high-quality trials supports the overall reliability of our findings.

## 5. Conclusions

Our meta-analysis demonstrates that, among carefully selected patients with ischemic stroke within the extended time window, IVT significantly improves excellent functional outcomes (mRS 0–1), with consistent benefits across thrombolytic agents (alteplase and tenecteplase), irrespective of the EVT status or imaging technique used. Additionally, IVT improved good functional outcomes (mRS 0–2), recanalization, reperfusion, and early neurological improvement. However, IVT was associated with an increased risk of sICH, particularly with alteplase and in patients not undergoing EVT. Importantly, no significant difference was observed in all-cause mortality or intervention-related mortality between the IVT and control groups. Unlike prior analyses, our study provides a comprehensive evaluation of both efficacy and safety outcomes stratified by key treatment modifiers. As all included trials used advanced imaging for patient selection, these findings apply specifically to advanced imaging-selected populations. These findings support the use of IVT as a potentially valuable therapeutic option for carefully selected patients in the extended window, with a need for ongoing vigilance regarding hemorrhagic risks. Future research should focus on refining patient selection criteria, determining the optimal dosing of thrombolytics, and directly comparing alteplase and tenecteplase to further enhance safety and efficacy.

## Figures and Tables

**Table 1:** Baseline characteristics of included studies.

Study	Year	Intervention	Sample Size		Age		Sex (M/F)		mRS Score		NIHSS Score		Treatment Time Window, h *		Occlusion Site		Endovascular Thrombectomy	
			Intervention	Control	Intervention	Control	Intervention	Control	Intervention	Control	Intervention	Control	Intervention	Control	Intervention	Control	Intervention	Control
EPITHET [9]	2014	Alteplase	38	31	75 (65–80)	73 (59–78)	23/15	19/12	0–2 (38)	0–2 (31)	12 (7.0–18.0)	11 (8.0–17.0)	4.5–6	4.5–6	NR	NR	None	None
WAKE-UP [22]	2018	Alteplase	254	249	65.3 ± 11.2	65.2 ± 11.9	165/89	160/89	0–1 (254)	0–1 (249)	6 (4–9)	6 (4–9)	≥4.5	≥4.5	ICA (24) MCA M1 segment (35) MCA M2 segment (32) Other (12)	ICA (11) MCA M1 segment (11) MCA M2 segment (37) Other (36)	None	None
ECASS-4 [23]	2019	Alteplase	61	58	76 (65–83)	79 (67–84)	36/25	31/27	0–1 (60)	0–1 (56)	10 (9)	9 (10)	4.5–9	4.5–9	NR	NR	None	None
EXTEND [24]	2019	Alteplase	113	112	73.7 ± 11.7	71.0 ± 12.7	59/54	66/46	0–1 (113)	0–1 (112)	12 (8.0–17.0)	10 (6.0–16.5)	4.5–9	4.5–9	ICA (12) MCA M1 segment (44) MCA M2 segment (25) MCA M3/4 segments (23) ACA (1) PCA (6) Other (2)	ICA (15) MCA M1 segment (38) MCA M2 segment (31) MCA M3/4 segments (18) ACA (2) PCA (4) Other (4)	None	None
THAWS [25]	2020	Alteplase	70	61	73.2 ± 12.4	75.8 ± 11.9	45/25	31/30	0–1 (70)	0–1 (61)	7 (4–13)	7 (5–12)	≥4.5	≥4.5	ICA (1) MCA M1 segment (6) MCA M2 segment (11) PCA (1)	ICA (2) MCA M1 segment (8) MCA M2 segment (11) BA (1)	None	None
TWIST [26]	2022	Tenecteplase	288	290	72.7 ± 11.3	72.9 ± 11.6	164/124	168/122	0 (188) 1 (63) 2 (37)	0 (191) 1 (57) 2 (42)	6 (5–11)	6 (5–10)	≥4.5	≥4.5	NR	NR	18 (6)	42 (14)
ROSE-TNK [27]	2023	Tenecteplase	40	40	62.68 ± 8.87	62.80 ± 8.56	31/9	26/14	0–1 (40)	0–1 (40)	7.5 (6–10.7)	7 (6–8.7)	4.5–24	4.5–24	Anterior circulation (24) Posterior circulation (16)	Anterior circulation (28) Posterior circulation (12)	None	None
TIMELESS [28]	2024	Tenecteplase	228	230	72 (62–79)	73 (63–82)	106/122	107/123	NR	NR	12 (8–17)	12 (8–18)	4.5–24	4.5–24	ICA (20) M1 segment (110) M2 segment (89) Other (9)	ICA (17) M1 segment (117) M2 segment (84) Other (12)	176 (77.2)	178 (77.4)
TRACE III [11]	2024	Tenecteplase	264	252	67 (58–75)	68 (59–76)	183/81	167/85	0 (230) 1 (34)	0 (216) 1 (36)	11 (7–15)	10 (7–14)	4.5–24	4.5–24	ICA (87) MCA M1 segment (119) MCA M2 segment (58)	ICA (84) MCA M1 segment (130) MCA M2 segment (38)	None **	None **
EXIT-BT [29]	2024	Tenecteplase	50	49	63.7 ± 9.6	64.8 ± 10.25	37/13	28/21	0–1 (50)	0–1 (49)	5 (4–7)	5 (4–6)	4.5–6	4.5–6	NR	NR	None	None
CHABLIS-T II [30]	2025	Tenecteplase	111	113	64.2 ± 10.4	63.6 ± 11.0	80/31	80/33	NR	NR	9 (5–14)	9 (6–16)	4.5–24	4.5–24	Extracranial segment of ICA (16) Intracranial segment of ICA (9) First segment of MCA (53) Second segment of MCA (21) ACA (10)	Extracranial segment of ICA (21) Intracranial segment of ICA (7) First segment of MCA (55) Second segment of MCA (24) ACA (6)	59 (53.2)	64 (56.6)
EXPECTS [10]	2025	Alteplase	117	117	64 (57–76)	63 (55–74)	75/42	78/39	0 (114) 1 (3)	0 (114) 1 (3)	3 (2–6)	3 (1–6)	4.5–24	4.5–25	NR	NR	None	None

Data are presented as mean ± SD, number of patients (n), or median (interquartile range). M, male; F, female; NR, not reported in the article; mRS, modified Rankin Scale; NIHSS, National Institutes of Health Stroke Scale. ACA, anterior cerebral artery; BA, basilar artery; ICA, internal carotid artery; PCA, posterior cerebral artery; MCA, middle cerebral artery. * Based on the time of symptom onset or last known well. ** None before randomization, rescue thrombectomy in four patients in the IVT group and five patients in the control group.

## Data Availability

All data generated or analyzed during this study are included in this article. Further inquiries can be directed to the corresponding author.

## Supplementary Materials

The following supporting information can be downloaded at https://www.mdpi.com/article/10.3390/diagnostics15141812/s1: Figure S1: Traffic light plot RoB 2 tool for risk of bias assessment; Figure S2: Summary plot RoB 2 tool for risk of bias assessment; Figure S3: Subgroup analysis of excellent functional outcome by imaging technique; Figure S4: Subgroup analysis of sICH by imaging technique; Figure S5: Leave-one-out analysis for reperfusion, excluding TIMELESS; Figure S6: Leave-one-out analysis for early neurological improvement, excluding EXPECTS; Figure S7: Leave-one-out analysis for early neurological improvement, excluding TRACE-III; Figure S8: Funnel plot for excellent functional outcome; Figure S9: Funnel plot for good functional outcome; Figure S10: Funnel plot for symptomatic intracranial hemorrhage (sICH); Figure S11: Funnel plot for 90-day all-cause mortality; Table S1: Search strategy; Table S2: Description of control arms in studies; Table S3: Certainty assessment; Table S4: Definitions of outcome measures across included studies
