# Supplementary material for: Efficacy and Safety of Intravenous Thrombolysis Beyond 4.5 Hours in Ischemic Stroke: A Systematic Review and Meta-Analysis
Source: Diagnostics (Basel). 2025 Jul 18;15(14):1812. doi: 10.3390/diagnostics15141812 (PMC12293326; doi:10.3390/diagnostics15141812)

Supplementary Appendix

Table S1: Search strategy

Database	Search string	Records
PubMed/MEDLINE	(((("ischemic stroke" OR stroke) AND ("thrombolysis" OR fibrinolysis OR "fibrinolytic therapy" OR tenecteplase OR alteplase OR tPA) AND ("beyond 4.5 hours" OR "extended time window" OR "late window" OR "at 4.5 to 24 hours" OR "4.5-24 hours" OR "up to 24 hours" OR "after 4.5 hours"))	766
Google Scholar	("ischemic stroke" OR "stroke") AND (thrombolysis OR tenecteplase OR alteplase OR tPA) AND ("after 4.5 hours" OR "late window" OR "extended time window") AND ("randomized controlled trial" OR RCT)	1450
Cochrane Library	("ischemic stroke" OR stroke) AND ("thrombolysis" OR fibrinolysis OR "fibrinolytic therapy" OR tenecteplase OR alteplase OR tPA) AND ("beyond 4.5 hours" OR "extended time window" OR "late window" OR "4.5 to 24 hours" OR "4.5-24 hours" OR "up to 24 hours" OR "after 4.5 hours")	85

Table S2: Definitions of outcome measures across included studies

Study	Recanalization	Reperfusion	Early neurological improvement	sICH
**EPITHET**	NR	>90% reduction in Tmax ≥ 2s lesion volume between baseline and day 3–5.	a reduction in NIHSS ≥8 points or reaching 0–1 at day 90.	Both National Institute of Neurological Disorders and Stroke (NINDS) and Safe Implementation of Thrombolysis in Stroke Monitoring Study (SITS–MOST) definitions
**WAKE-UP**	NR	NR	NR	SITS–MOST definition: local or remote parenchymal hematoma type 2 on the imaging scan obtained 22 to 36 hours after treatment, plus neurologic deterioration, as indicated by a score on the NIHSS that was higher by 4 points or more than the baseline value or the lowest value between baseline and 24 hours, or hemorrhage leading to death.
**ECASS-4**	NR	NR	NR	defined as any apparently extravascular blood in the brain or within the cranium that was associated with clinical deterioration, as

				defined by an increase of 4 points or more in the score on the NIHSS, or that led to death and that was identified as the predominant cause of the neurologic deterioration.
**EXTEND**	a score of 2 or 3 on the Arterial Occlusive Lesion scale [range, 0 to 3], indicating partial or complete opening of the artery, respectively, with the presence of distal blood flow in the patients who had occlusion of a cerebral vessel detected on CT or MR angiography at baseline	≥50% and ≥90% reductions, respectively, in the volume of the perfusion lesion in which there had been a delayed arrival of an injected tracer agent exceed ing 6 seconds	a reduction in the NIHSS score of ≥8 points or a score of 0 or 1 within 24 hours, 72 hours, and 90 days after the intervention.	parenchymal hematoma type 2 (confluent blood clot occupying >30% of the infarct with substantial mass effect) within 36 hours after intervention, accompanied by an increase of at least 4 points in the NIHSS score from baseline
**THAWS**	defined by modified Mori grade 3.	NR	NR	an increase in NIHSS score by ≥4 from baseline and parenchymal hematoma type II on MRI at 22 to 36 hours after initiation of treatment.
**TWIST**	NR	NR	NR	SITS-MOST and IST-3 definitions: Local or remote parenchymal haematoma type 2 on the imaging scan obtained 22–36 h after treatment plus neurological

				deterioration (indicated by NIHSS score that was higher by 4 points or higher than the baseline value or the lowest value between baseline and 24 h, or haemorrhage leading to death). Clinically significant deterioration (neurological deterioration; new headache, acute hypertension, nausea, or vomiting; or a sudden decrease in consciousness) or death within the first 7 days of treatment with evidence of substantial brain parenchymal haemorrhage (local or distant from the infarct) or substantial haemorrhagic transformation of an infarct on brain imaging.
**ROSE-TNK**	NR	NR	more than 4-point decrease in NIHSS score within 24 hours.	an increase in the NIHSS score of ≥4 points because of the intracranial hemorrhage
**TIMELESS**	defined as complete recanalization (arterial occlusive lesion score, 3; scale range, 0 [no recanalization]	more than 90% reduction in the penumbra, as estimated from the volume of tissue for which there was delayed arrival	NR	an increase of atleast 4 points in the NIHSS score, as compared with the most recent NIHSS score, that was attributed to bleeding on CT

	to 3 [complete recanalization]).	of an injected tracer agent (time to maximum of the residue function) exceeding 6 seconds, between baseline and 24-hour perfusion imaging.		(preferred) or MRI performed within 36 hours after the receipt of tenecteplase or placebo.
**TRACE III**	assessed with the use of the Arterial Occlusive Lesion scale (scores range from 0 [no recanalization] to 3 [complete recanalization]).	a reduction of greater than 90% in the volume of the lesion in which there had been a delayed arrival of an in jected tracer agent exceeding 6 seconds.	an improvement (reduction) from baseline of at least 8 points on the NIHSS or an NIHSS score 1 or lower.	defined according to the criteria established ECASS III trial: the presence of any extravascular blood in the brain or within the cranium that was associated with clinical deterioration, as defined by an increase of 4 points or more in the score on the NIHSS, or that led to death and that was identified as the predominant cause of the neurologic deterioration.
**EXIT-BT**	NR	NR	more than 4 point decrease in NIHSS within 24 h	an increase in the NIHSS score of ≥ 4 points as a result of the intracranial hemorrhage
**CHABLIS-T II**	a score ≥ 2 on the Thrombolysis in Myocardial Infarction (TIMI) scale of at the 4- to 6- hour CTA (reconstructed from CTP) for patients not transferred to	restoration of blood flow of >50% of the involved ischemic territory.	≥8-point reduction in the NIHSS or an NIHSS score of 0–1	any type of intracranial hemorrhage with at least 4 points increase in the NIHSS score from baseline.

	angio-suite, or at first angiographic acquisition prior to thrombectomy for patients transferred to angio-suite			
**EXPECTS**	NR	NR	a reduction of ≥8 points on the NIHSS or an NIHSS score of ≤1	any ICH associated with a worsening [i.e., increase] of ≥4 points on the NIHSS or leading to death that was identified as the predominant cause of neurologic deterioration within 36 hours after randomization

Table S3: Ceratainty assessment for Intravenous thrombolysis beyond 4.5 hours in ischemic stroke

Certainty assessment						
Participants (studies) Follow-up	Risk of bias	Inconsistency	Indirectness	Imprecision	Publication bias	Overall certainty of evidence
**Excellent functional outcome**						
3208<br>(12 RCTs)	not serious	not serious	not serious	not serious	none	⨁⨁⨁⨁<br>High
**Good functional outcome**						
3221<br>(12 RCTs)	not serious	not serious	not serious	not serious	none	⨁⨁⨁⨁<br>High
**Recanalization**						
1349<br>(5 RCTs)	not serious	not serious	not serious	not serious	undetected	⨁⨁⨁⨁<br>High
**Reperfusion**						
1330<br>(5 RCTs)	not serious	serious	not serious	not serious	undetected	⨁⨁⨁◯<br>Moderate
**Early neurological improvement**						
1356<br>(6 RCTs)	not serious	not serious	not serious	not serious	undetected	⨁⨁⨁⨁<br>High
**sICH**						
3125<br>(11 RCTs)	not serious	not serious	not serious	not serious	none	⨁⨁⨁⨁<br>High
**90-day all-cause mortality**						
2903<br>(10 RCTs)	not serious	not serious	not serious	serious	none	⨁⨁⨁◯<br>Moderate

Any ICH

2179<br>(9 RCTs)	not serious	not serious	not serious	not serious	undetected	⨁⨁⨁⨁<br>High

Type II PH

2432<br>(7 RCTs)	not serious	not serious	not serious	not serious	undetected	⨁⨁⨁⨁<br>High

Systemic hemorrhage

2356<br>(8 RCTs)	not serious	not serious	not serious	serious	undetected	⨁⨁⨁◯<br>Moderate

7-day all-cause mortality

341<br>(2 RCTs)	not serious	not serious	not serious	serious	undetected	⨁⨁⨁◯<br>Moderate

90-day intervention-related mortality

421<br>(3 RCTs)	not serious	not serious	not serious	serious	undetected	⨁⨁⨁◯<br>Moderate

Table S4: Description of control arms in studies

**Study**	**Control**
EPITHET 2014	Placebo
WAKE-UP 2018	Placebo
ECASS-4 2019	Placebo
EXTEND 2019	Placebo
THAWS 2020	Standard medical care*
TWIST 2022	Standard medical care*
ROSE-TNK 2023	Standard medical care*
TIMELESS 2024	Placebo
TRACE III 2024	Standard medical care*
EXIT-BT 2024	Standard medical care* and NBP 50 mg daily
CHABLIS-T II 2025	Best medical treatment (excluding tenecteplase)
EXPECTS 2025	Standard medical treatment**

*Standard medical care was defined as antiplatelets, statins, blood pressure and glucose control, and general supportive care according to European or national guidelines for acute stroke.

**Standard treatment group received antiplatelet therapy and other treatments in accordance with the Chinese Guidelines for Diagnosis and Treatment of Acute Ischemic Stroke 2018.

Figure S1: Traffic light plot RoB 2 tool for risk of bias assessment

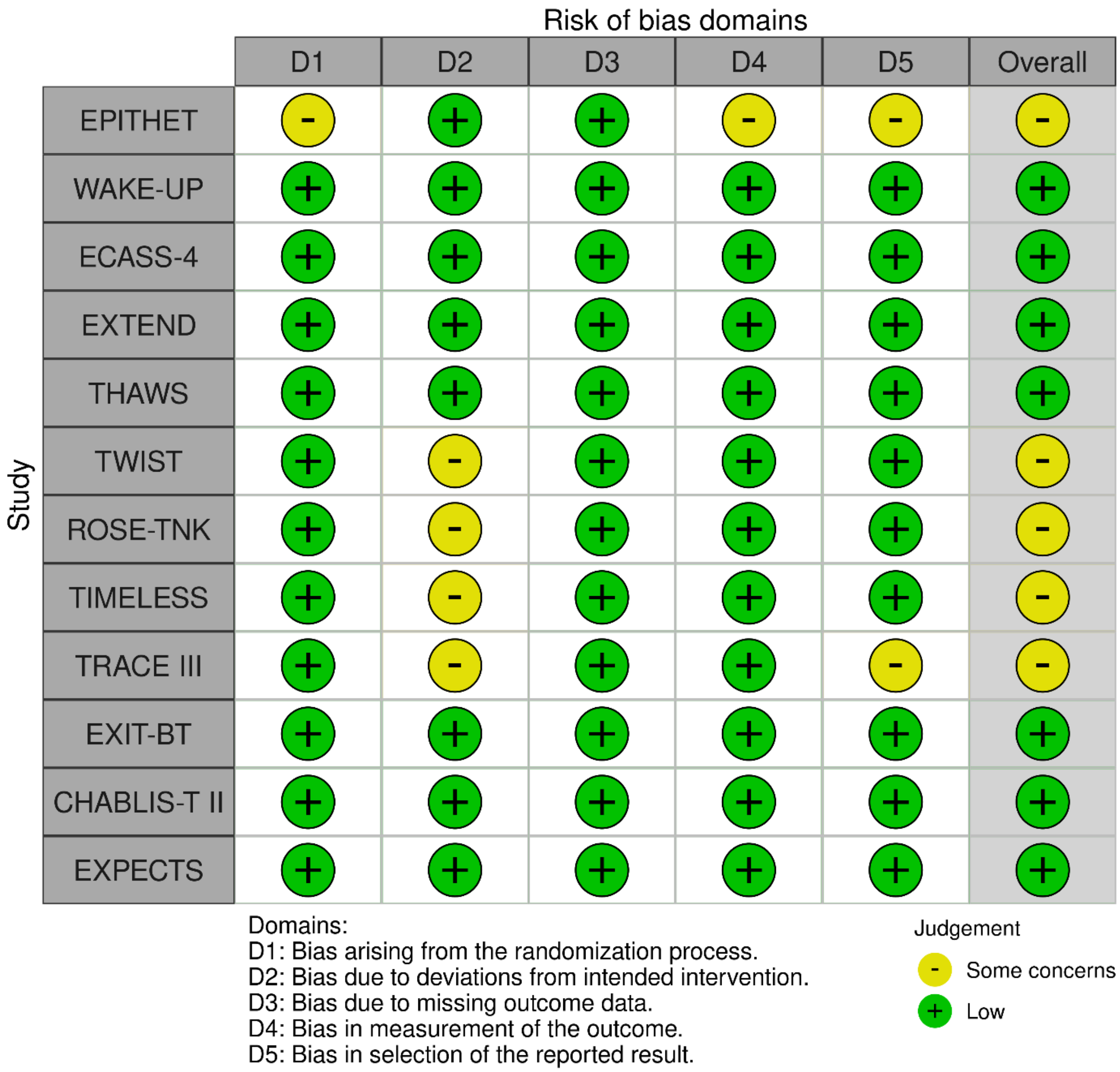

Figure S2: Summary plot RoB 2 tool for risk of bias assessment

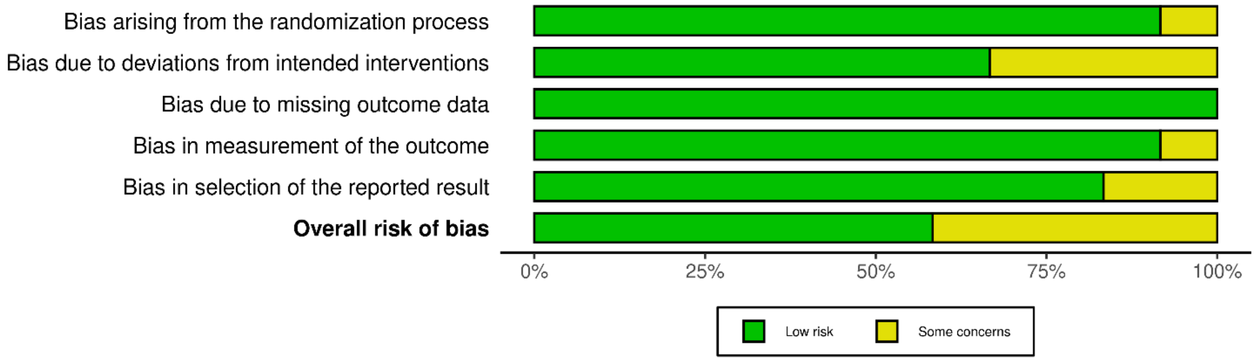

Figure S3: Subgroup analysis of Excellent Functional Outcome by imaging technique

Study or Subgroup	IVT Events	IVT Total	Control Events	Control Total	Weight	Odds Ratio IV, Random, 95% CI
**1.16.1 DWI-FLAIR mismatch**						
ROSE-TNK 2023	21	40	20	40	4.0%	1.11 [0.46, 2.66]
THAWS 2020	32	68	28	58	6.3%	0.95 [0.47, 1.92]
WAKE-UP 2018	131	246	102	244	24.4%	1.59 [1.11, 2.27]
**Subtotal (95% CI)**		**354**		**342**	**34.8%**	**1.39 [1.03, 1.87]**
Total events	184		150			
Heterogeneity: Tau² = 0.00; Chi² = 1.90, df = 2 (P = 0.39); I² = 0%						
Test for overall effect: Z = 2.14 (P = 0.03)						
**1.16.2 MRI or CT perfusion**						
CHABLIS-T II 2025	44	111	41	113	10.7%	1.15 [0.67, 1.98]
ECASS-4 2019	21	60	16	56	5.0%	1.35 [0.61, 2.95]
EXTEND 2019	40	113	33	112	9.9%	1.31 [0.75, 2.30]
TIMELESS 2024	72	226	59	229	18.7%	1.35 [0.90, 2.02]
TRACE III 2024	87	264	61	252	20.9%	1.54 [1.05, 2.26]
**Subtotal (95% CI)**		**774**		**762**	**65.2%**	**1.36 [1.10, 1.70]**
Total events	264		210			
Heterogeneity: Tau² = 0.00; Chi² = 0.77, df = 4 (P = 0.94); I² = 0%						
Test for overall effect: Z = 2.79 (P = 0.005)						
**Total (95% CI)**		**1128**		**1104**	**100.0%**	**1.37 [1.15, 1.64]**
Total events	448		360			
Heterogeneity: Tau² = 0.00; Chi² = 2.68, df = 7 (P = 0.91); I² = 0%						
Test for overall effect: Z = 3.52 (P = 0.0004)						
Test for subgroup differences: Chi² = 0.01, df = 1 (P = 0.94), I² = 0%						

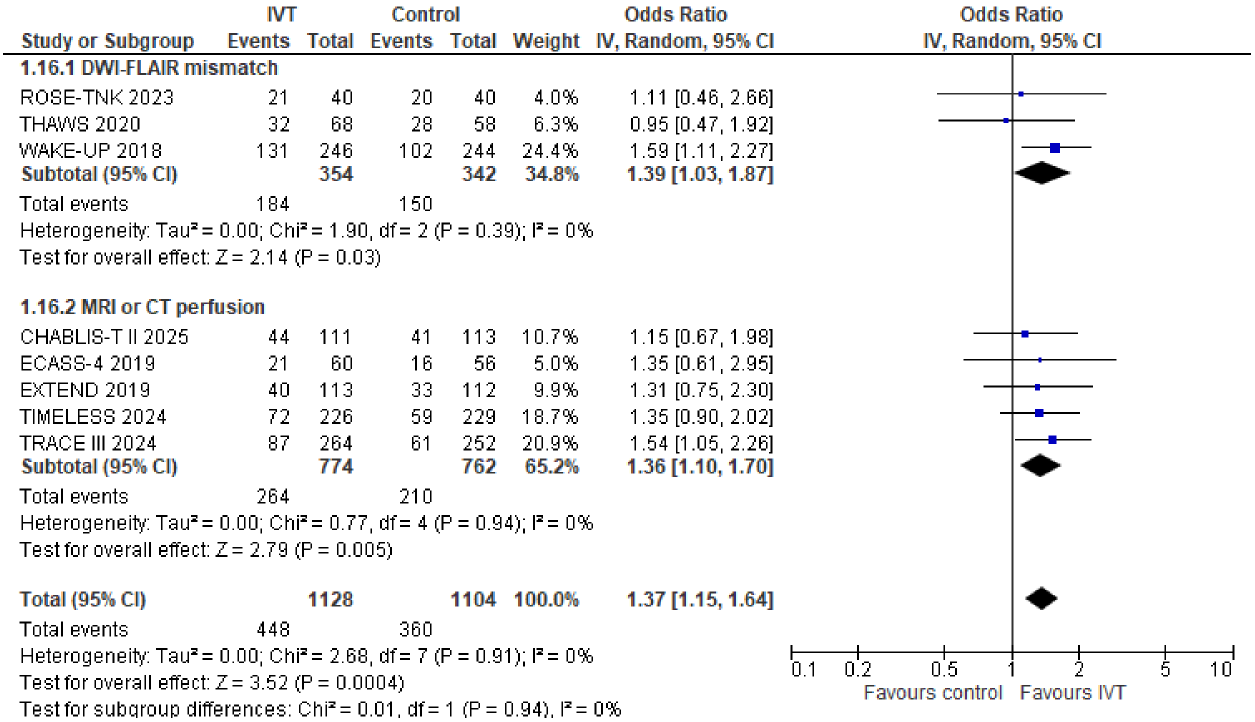

Figure S4: Subgroup analysis of sICH by imaging technique

Study or Subgroup	IVT Events	IVT Total	Control Events	Control Total	Weight	Odds Ratio IV, Random, 95% CI
**1.17.1 DWI-FLAIR mismatch**						
ROSE-TNK 2023	0	40	0	40		Not estimable
THAWS 2020	1	71	0	60	3.9%	2.57 [0.10, 64.37]
WAKE-UP 2018	5	251	1	244	8.8%	4.94 [0.57, 42.59]
**Subtotal (95% CI)**		**362**		**344**	**12.7%**	**4.04 [0.67, 24.19]**
Total events	6		1			
Heterogeneity: Tau² = 0.00; Chi² = 0.11, df = 1 (P = 0.74); I² = 0%						
Test for overall effect: Z = 1.53 (P = 0.13)						
**1.17.2 MRI or CT perfusion**						
CHABLIS-T II 2025	6	111	5	113	27.5%	1.23 [0.37, 4.17]
ECASS-4 2019	1	60	0	56	3.9%	2.85 [0.11, 71.39]
EXTEND 2019	7	113	1	112	9.1%	7.33 [0.89, 60.59]
TIMELESS 2024	7	218	5	214	30.1%	1.39 [0.43, 4.44]
TRACE III 2024	8	264	2	252	16.7%	3.91 [0.82, 18.58]
**Subtotal (95% CI)**		**766**		**747**	**87.3%**	**2.00 [1.01, 3.97]**
Total events	29		13			
Heterogeneity: Tau² = 0.00; Chi² = 3.19, df = 4 (P = 0.53); I² = 0%						
Test for overall effect: Z = 2.00 (P = 0.05)						
**Total (95% CI)**		**1128**		**1091**	**100.0%**	**2.19 [1.16, 4.14]**
Total events	35		14			
Heterogeneity: Tau² = 0.00; Chi² = 3.81, df = 6 (P = 0.70); I² = 0%						
Test for overall effect: Z = 2.41 (P = 0.02)						
Test for subgroup differences: Chi² = 0.51, df = 1 (P = 0.47), I² = 0%						

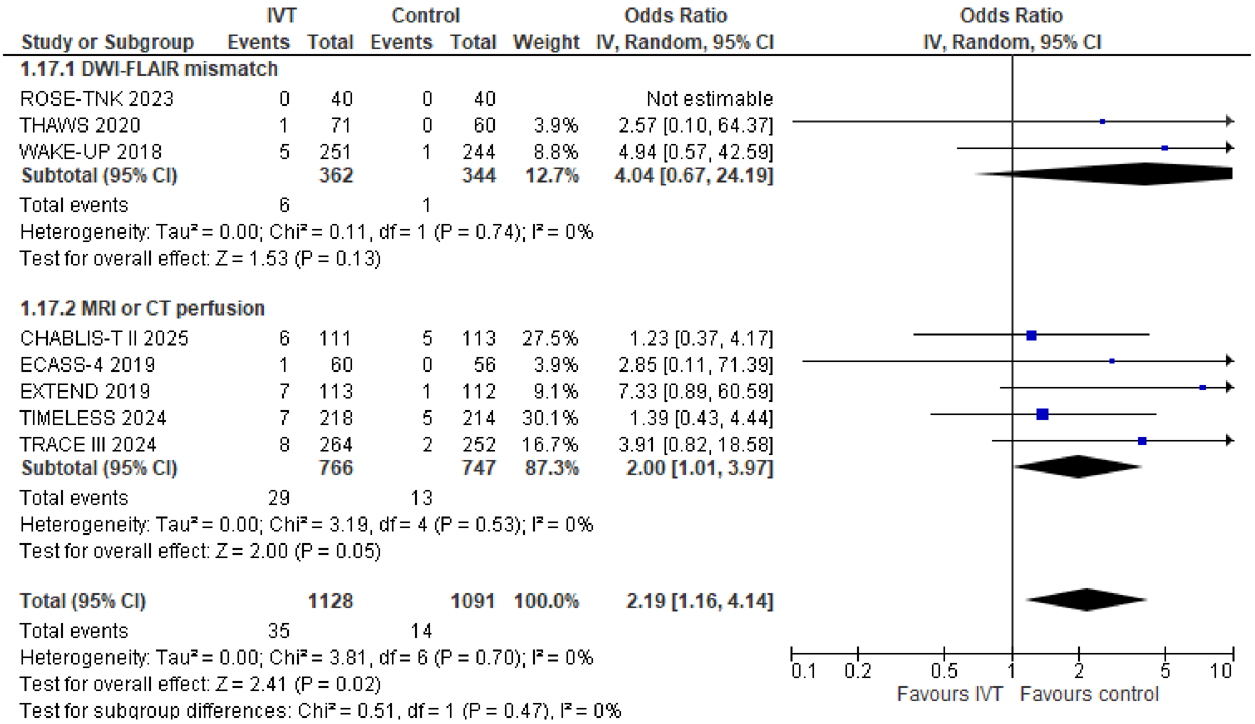

Figure S5: Leave-one-out analysis for reperfusion, excluding TIMELESS

Study or Subgroup	IVT Events	IVT Total	Control Events	Control Total	Weight	Odds Ratio IV, Random, 95% CI
CHABLIS-T II 2025	37	111	12	111	21.4%	4.13 [2.01, 8.45]
EPITHET 2014	22	38	8	31	11.5%	3.95 [1.41, 11.08]
EXTEND 2019	53	106	31	109	31.2%	2.52 [1.43, 4.42]
TRACE III 2024	48	239	27	229	35.9%	1.88 [1.13, 3.14]
**Total (95% CI)**		**494**		**480**	**100.0%**	**2.65 [1.84, 3.83]**
Total events	160		78			

Heterogeneity: $Tau^2 = 0.03$; $Chi^2 = 3.78$, $df = 3$ ($P = 0.29$); $I^2 = 21\%$

Test for overall effect: $Z = 5.21$ ($P < 0.00001$)

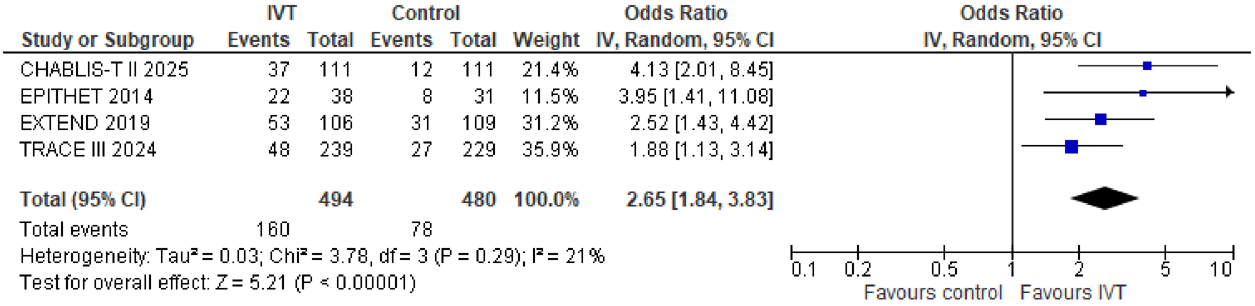

Figure S6: Leave-one-out analysis for early neurological improvement, excluding EXPECTS

	IVT		Control			Odds Ratio
Study or Subgroup	Events	Total	Events	Total	Weight	IV, Random, 95% CI
CHABLIS-T II 2025	24	110	26	112	25.4%	0.92 [0.49, 1.73]
EXIT-BT 2024	10	49	2	49	10.3%	6.03 [1.25, 29.15]
EXTEND 2019	41	112	25	112	26.3%	2.01 [1.12, 3.62]
ROSE-TNK 2023	11	40	3	40	12.5%	4.68 [1.19, 18.34]
TRACE III 2024	40	250	15	249	25.5%	2.97 [1.60, 5.53]
**Total (95% CI)**		**561**		**562**	**100.0%**	**2.27 [1.24, 4.14]**
Total events	126		71			

Heterogeneity: Tau² = 0.27; Chi² = 10.71, df = 4 (P = 0.03); I² = 63%

Test for overall effect: Z = 2.67 (P = 0.008)

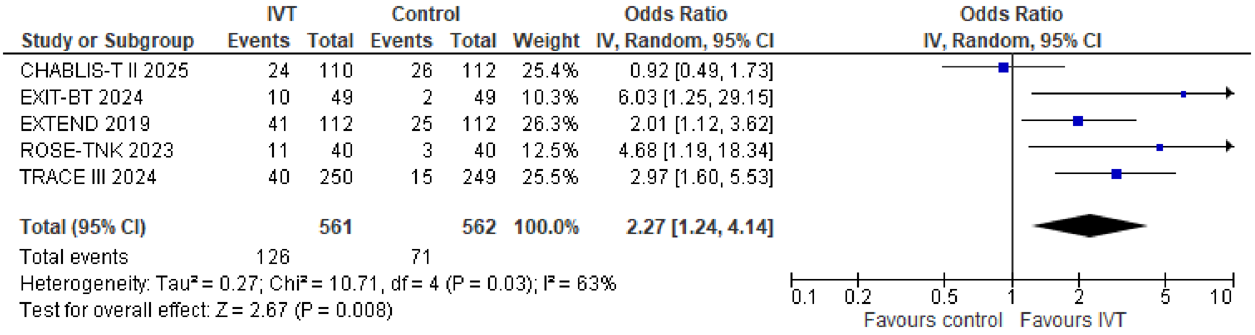

Figure S7: Leave-one-out analysis for early neurological improvement, excluding TRACE-III

	IVT		Control			Odds Ratio
Study or Subgroup	Events	Total	Events	Total	Weight	IV, Random, 95% CI
CHABLIS-T II 2025	24	110	26	112	25.0%	0.92 [0.49, 1.73]
EXIT-BT 2024	10	49	2	49	9.9%	6.03 [1.25, 29.15]
EXPECTS 2025	42	116	42	117	27.2%	1.01 [0.59, 1.73]
EXTEND 2019	41	112	25	112	26.0%	2.01 [1.12, 3.62]
ROSE-TNK 2023	11	40	3	40	12.0%	4.68 [1.19, 18.34]
**Total (95% CI)**		**427**		**430**	**100.0%**	**1.69 [0.95, 3.04]**
Total events	128		98			

Heterogeneity: Tau² = 0.25; Chi² = 10.82, df = 4 (P = 0.03); I² = 63%
Test for overall effect: Z = 1.77 (P = 0.08)

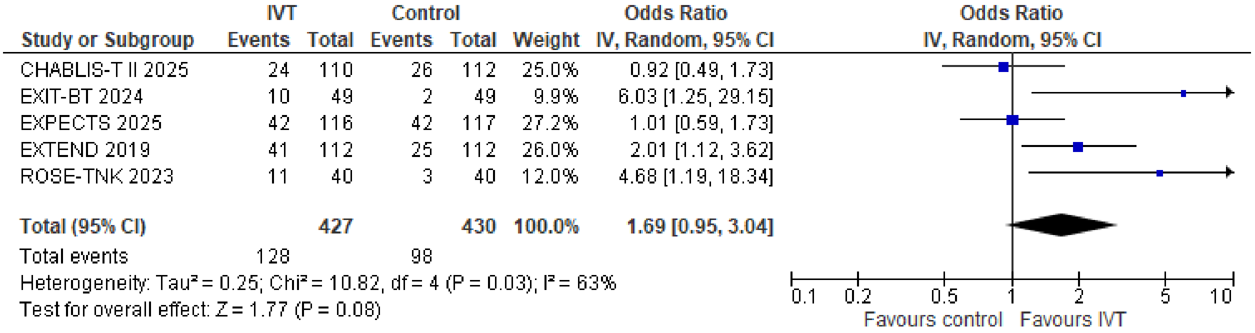

Figure S8: Funnel plot for excellent functional outcome

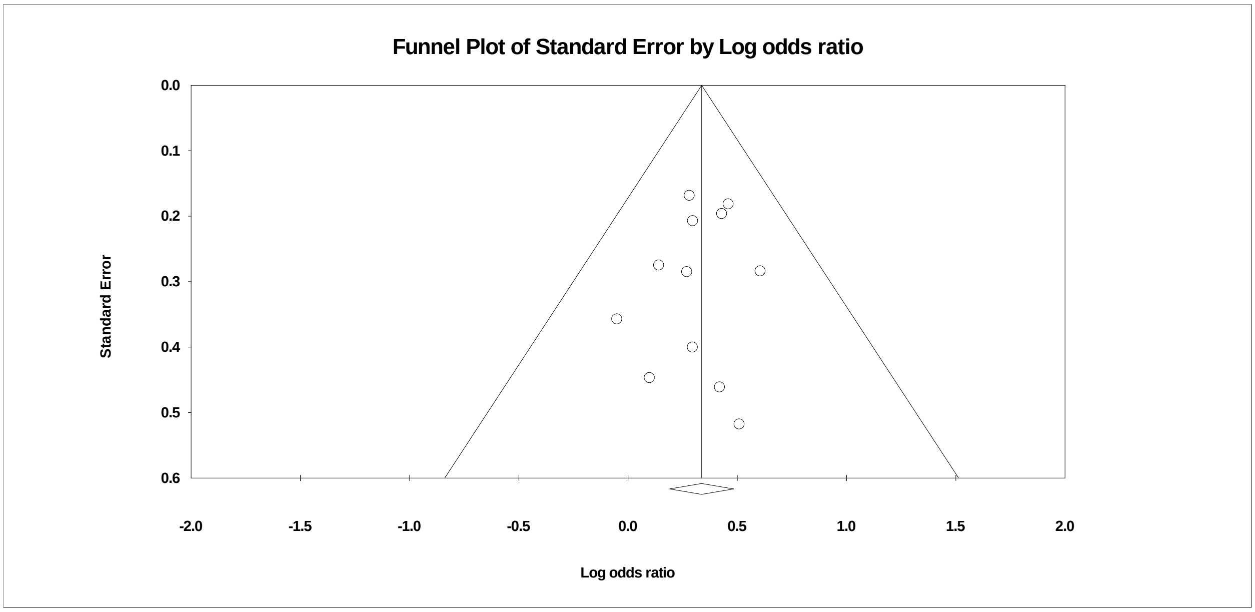

Figure S9: Funnel plot for good functional outcome

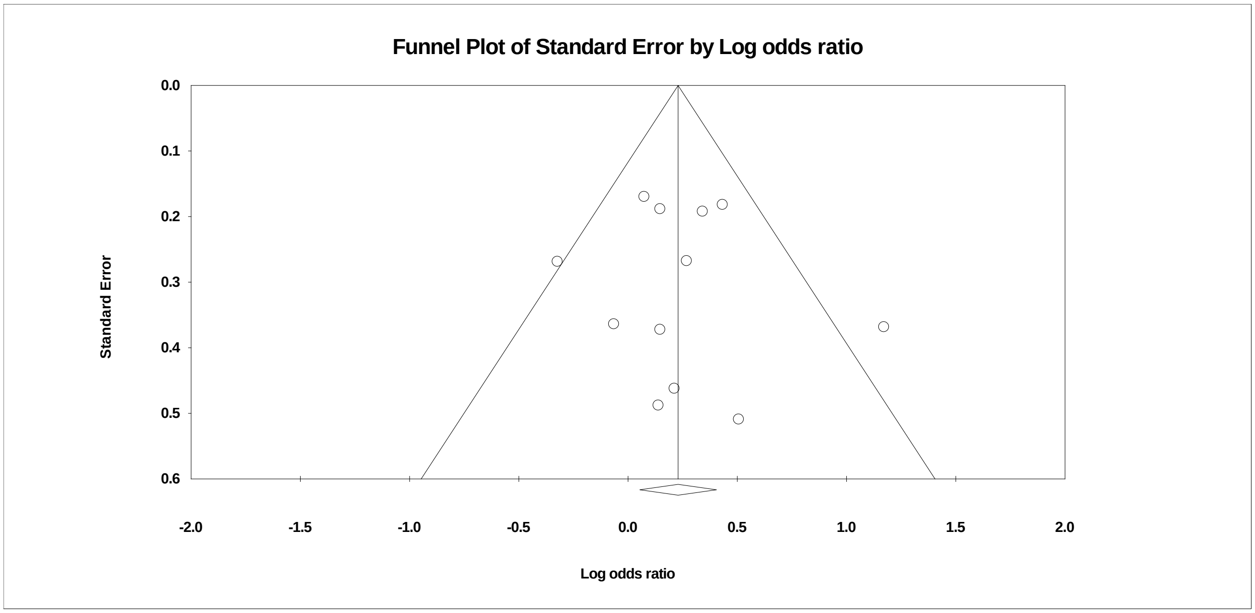

Figure S10: Funnel plot for symptomatic intracranial hemorrhage (sICH)

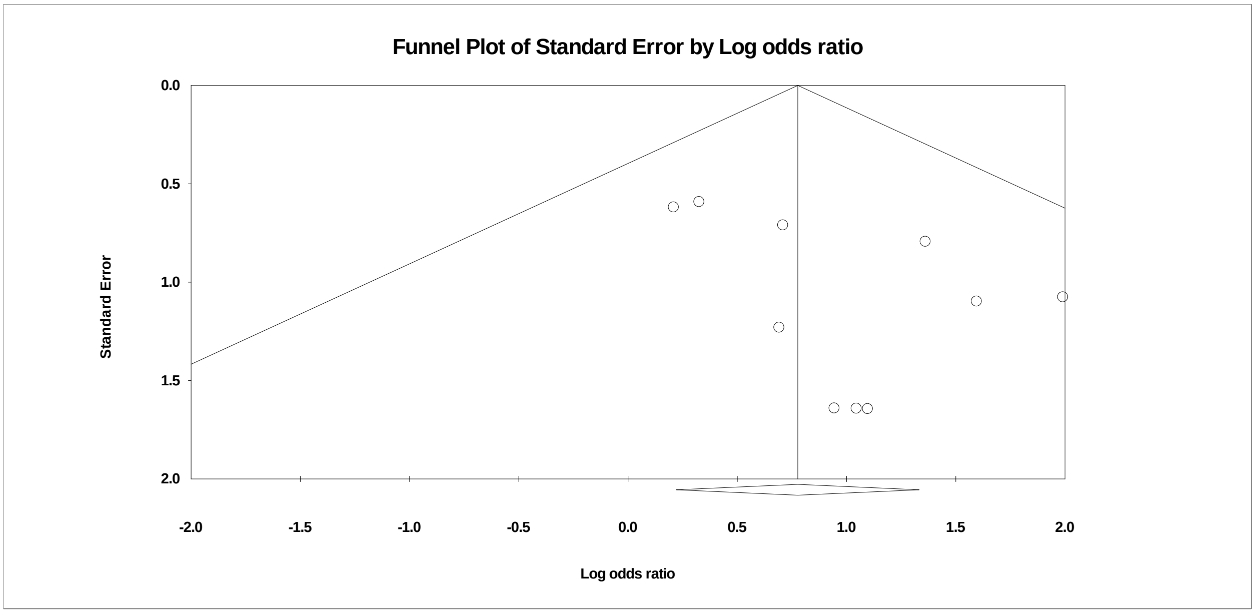

Figure S11: Funnel plot for 90-day all-cause mortality

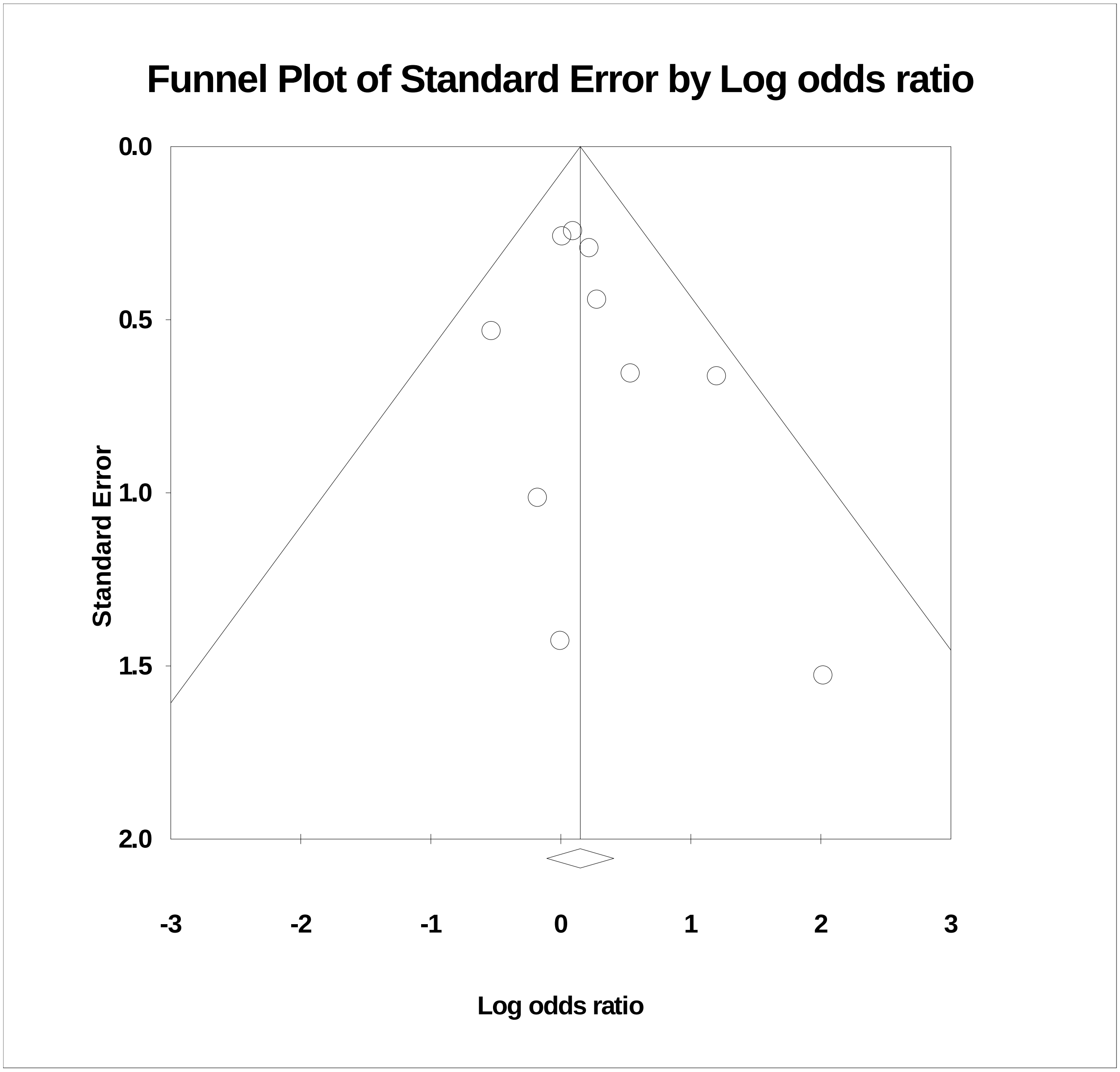

Supplement: Supplementary file 1 [file diagnostics-15-01812-s001.zip › diagnostics-3685323-supplementary.pdf]
